# Extra-Limites

**Published:** 1844-01-01

**Authors:** 


### EXTRA-LIMITES.

### INDIAN MEDICAL SERVICE.

*India, 20th August*, 1843.

Sir,—With reference to an attack lately made in the Medico-Chirurgical Review on the character of the Indian Medical Service, and the recommendation of Messrs. Annesley and Martin to abolish the Medical Board, &c., permit me to call your attention to the articles marked with a cross in the newspapers I take the liberty of sending you. I shall feel much obliged if you will devote one half-hour of your valuable time to them, because they express both the opinion of the public of India, and the sentiments of all or a great majority of the medical men who are still in the East India Company's Service. The connexion with it of those two gentlemen having ceased, it is little creditable to the better feelings of our nature, that they should suggest measures opposed to the interests of a body of which they were lately members. It now appeais that to the importunities of Mr. Martin we have to ascribe the regulations which have completely changed the constitution of the medical department. Before they came into operation, the step of Superintending Surgeon with the comparative rank of Lieutenant-Colonel was attained in the Bombay Presidency in 22 or 23 years, and the average of retiring from the Board on the highest pension with the rank of Brigadier-General was 30 years. The Superintending Surgeoncy is now declared to be a staff appointment, with no higher commission than that of Surgeon—the relative rank of Major, *without the emoluments*, being in lieu assigned to "Senior Surgeons" of 30 years' service. Our claim to promotion by seniority, one of the most firmly established principles of the Company's Service, and one which made it the most desirable for a man without interest to enter, is set aside: the period for retiring on the highest pension is raised from 30 to 38 or 41 years, according to the number of furloughs bad health may have compelled a man to take, 35 years' actual service in India being exacted: pensions according to rank are abolished, though military officers retain all the privileges of the old regulations as well as those of "the Boon:" and the 2nd rate of pension (£300.) is given eight years after that of Captain, whereas military officers obtain that of Major (£292.) four years after they have made out their title to that of Captain. The progressive scale of pensions will no doubt tempt men to protract their stay from term to term till they are either summoned to another world, or become entitled to the highest at the age of 63 or 66—the average on arrival not being less than 25. Man's days are limited to threescore years and ten, but according to the rule that every healthy adult will live half the difference between his own age and 81, the expectation of life of Assistant-Surgeons on their arrival in India, even with a European tenure of it, is only 28, so that in all human probability we shall have been at least 10 years in our graves when the time comes at which we would if living be entitled to the highest rate of pension. How many years will it be enjoyed by the very very few (one out of 600 of those who enter the Service, according to Badenack) who live to be entitled to it, and how differently is life in India valued by Insurance Societies, which demand more than double premium?

To a seat at the Medical Board we all look forward, if our lives be spared, as a reward for long and faithful service, for the prizes we can aspire to are scattered with a sparing hand. The Military branch has about 13 per cent. of field officers; that is, every regiment of 23 officers has one Colonel, one Lieut.-Colonel, and one Major, below which latter rank very few indeed have retired since permission was given to accept a bonus for retiring, and that bonus in the case of Majors seldom falls short of 30,000 rupees in addition to their pension. The Indian Navy, consisting of 138 officers, has also 13 per cent., there being 18 above the rank of Naval Lieutenant. The Medical branch had 4⅛ per cent., but the former rank of the Superintending Surgeons being now taken away, the whole commissioned Medical Establishment of India, numbering upwards of 800 members, supernumeraries included, has only nine officers holding commissions higher than that of a Captain. In this country relative military rank is assigned not only to the Navy, but to the civil and clerical services, civil servants of four years' standing ranking with Captains, of eight with Majors, of 12 with Lieut.-Colonels, of 20 with Colonels, &c. Medical officers have the rank of Major after 30!!! They are thus placed at the bottom of the class of gentlemen, and they alone, of all the Company's servants, who in general are sprung from the middle classes at home, acquire by their appointments no elevation of social position till they have been 30 years in India, for the title of Esquire, which is equal to the rank of Captain, is conceded to all medical men in civil life. No medical man has ever had a seat in the India Direction, and to this is to be attributed our humble influence. After the capture of Ghuznee, where honors were showered in profusion on the army of the Indus, Dr. Kennedy, not only the senior superintending surgeon, but the senior in rank of all the staff officers, was not even decorated with the third class of the Doorance Order, though his zeal and talents drew forth the highest compliment. The chief medical officer, and the chief commissariat officer were named together in terms of equal commendation in the same paragraphs of Sir Charles Napier's despatches after the battles of Meeanee and Hydrabad. The former is unhonoured and unnoticed by Government; the latter is promoted from Captain to Major, and made a Companion of the Bath. Such painful distinctions not only detract from the respect due to the India Medical Officers, but through them derogate from the honor and dignity of the whole profession.

These facts must be well known to Messrs. Annesley and Martin, and yet they advise the abolition of the Medical Board, which, depriving us of the few remaining appointments that give a higher rank than that of Surgeon, would be a blow very little lighter than the one we have already sustained at their instigation. Let us next inquire what is to be gained by such a measure? Instead of three members with good salaries, and a Secretary with a large establishment of Clerks at each Presidency, there would be one Director-General with a large salary, and three Secretaries with small ones. The Secretaries would constitute the Board in the management of all ordinary business, for it is impossibe that in the relaxing climate of the Presidencies the health of one man can at all times be equal to such duties as the Board has to perform. The Home Government may say "detur digniori," and resolve that the Director-Generalship shall be given on this principle, but it is probable enough that the Governor-General, even if he relinquish all rights of patronage, and disregard all claims of friendship, may pass over men who from seniority have the best right to preferment, and select one who is by no means the best qualified. This under the new regulations will no doubt be the case with members of the Board and Superintending Surgeons, even if qualifications are considered of any consequence in comparison with interest, which commands every appointment; and injustice will be done to men who entered the service on the faith of the permanency of the system of promotion by seniority. Even if a man of pre-eminent talents and zeal be occasionally selected, it is not likely that, for the five years he can hold office, he will have the interest of the service more at heart than the present members, who at all events are men of average talents and acquirements. Boards have been found most efficient in the management of other affairs, for there is a Government Council Board, a Revenue Board, a Military Board, a Clothing Board, &c., and it is strange that a Board, of which the President, who is now designated Physician-General, is the responsible member, should be incapable of managing medical affairs. It does not necessarily follow because under such a direction the medical affairs of the Royal Army did not prosper, that the medical service of India requires a Director-General. The Medical Board in London was composed of men who never served beyond the limits of the metropolis. The Boards in India consist of men who have been trained to the superintendence and direction of the medical concerns of the army from the day they entered it, and they have never been convicted of inefficiency. The Lancet, it is true, lately imputed to the Medical Board of Calcutta remissness in not making complete medical and scientific returns, and in consequence accused it of "deplorable incapacity and apathy." The whole charge originating in the wilful ignorance of the Editor of the Lancet, to whom Journals publishing the very reports he bewails the want of had been sent, and Major Tulloch's neglecting to make application in the proper quarter, was fully and clearly refuted in the India Medical Journal for November, 1842.

You have long been looked up to as the highest authority in the profession, and you have ever been the defender of our rights; I would therefore, with all confidence—though your pages have lately been lent to our disparagement, to please one or two dissatisfied, perhaps disappointed individuals—still commit the advocacy of our cause to your hands.

I remain, Sir,
Your most obedient, humble Servant,
One of the 800.

To Dr. James Johnson,

*Suffolk Place, Pall-mall East, London.*

---

### THE KHEESAH, or INDIAN FLESH GLOVE.

Although the importance of the condition of the Skin to the maintenance of health and the comfort of the individual has been fully demonstrated by the writings of many eminent physiologists and physicians, yet few modes in this country have hitherto been devised either to promote or to regulate its functions. The Skin is not to be looked upon merely as an elastic covering, serving to protect the parts beneath it from injury, and to preserve the symmetry and form of the body; but as ministering to the sense of feeling, and to some of the most important offices of the system. The Skin is different in its degrees of thickness in different parts, and from it issue various peculiar secretions by many, almost innumerable orifices, which admit of the exit of the perspiration from the body. The minuteness of these pores is evident from the exhalation which they give forth, being of so delicate a character as to be discharged in the form of vapour, and thus constituting what is commonly known as the *Insensible Perspiration.* In another state this is condensed, and forms the *Sensible Perspiration,* the secretion of which, is known often to be most profuse and rapid. By means of this secretion, that which is hurtful to the body within, is freely thrown off, and the skin thus constitutes one of the most effectual preservers of the frame in the discharge of irritating and injurious matters. Lymphatic vessels also in great number run upon the surface of the Skin, and imbibe various materials which they convey into the body. The absorbing power of the Skin exists to a very great degree.

Without going into particulars respecting these several functions of the Skin, it will be apparent, that to preserve the surface free from all extraneous substances, to dislodge all concreted matter, collected dust, the deposit of the fatty secretions, &c., it must be of the utmost importance to use those means of ablution, friction, &c., which in countries, where the temperature of the atmosphere is high, are known to be attended with such beneficial results. Frictions not of a *violent,* but of a *gentle* nature, are universally practised by the natives of the East for the purposes above stated, as a substitute for exercise, and means have been adopted by them to effect this purpose in the most perfect manner. The most effectual application resorted to in India is a Glove made of the

BURRUK, or Persian Glove Cloth, called the KHEESAH, or INDIAN FLESH GLOVE, which, at a considerable cost and trouble, Messrs. SAVORY and MOORE are enabled to present to the public. The KHEESAH has been in use from time immemorial in the Empire of Hindustan, Persia, and throughout the East; countries in which the greatest attention has always been paid to the purity, softness, and polish of the Skin.

It is applicable alike to the Bath and the Dressing Room; and, unlike the hair-glove, which in India is used only for rubbing down horses, it rouses the activity of the skin, removes all impurities, and elicits an agreeable and equable action towards the surface, and that without occasioning the smallest discomfort or irritation.

This superior article of comfort, health, and luxury comes thus recommended to us, not only by the opinion and experience of Tropical Physicians, but through the experience of ages, amongst a people, who of all nations have paid most attention to maintaining the natural functions of the Skin.

The INDIAN FLESH-GLOVE will be found suited to the most delicate, as well as to the coarsest Skins, simply requiring, in the latter instance, a little more power in using the friction;—indeed, so ingeniously is the texture of this Glove framed, that the two sides of it are differently constructed, so as to be adapted to different Skins. It thus possesses unequalled advantages.

136, New Bond Street, and 220, Regent Street, London.

### PENNY DOCTORS.

THE aristocratic constitution of the profession does not please some people. They would have it more on the equality principle. We dare say they are right, and as they assure us that things are rapidly, or, at all events, certainly going on that way, we may some of us live to enjoy it. In the interim we cannot but esteem those pioneers of civilisation who are a-head of their age, and anticipate the future.

A gentleman of this description, who rejoices in the name of *Funge*, or, perhaps, *Fudge*, has arisen in Cheltenham. His *affiche* speaks for itself. Here it is:—

"*The Cheltenham Penny Medical and Surgical Society, established for the especial Benefit of Servants and Working Classes, and conducted by Mr. W. C. Funge, Surgeon, Apothecary, and Accoucheur, at his residence, No.* 2, *Buckingham Villas, Wellington Street.—Hours of attendance, daily (Sundays excepted), mornings, from nine to twelve; evenings, from six to seven.*

"RULES.

"1. A subscription of one penny per week (to be paid every Monday), entitles the subscriber to medical and surgical advice, and medicine, so long as the same is paid.

"2. Subscribers requiring medical or surgical advice, &c. (in all cases that will admit), to attend upon the surgeon within the hours above specified.

"3. Subscribers requiring medical or surgical advice, &c., whose case will not admit of attending upon the surgeon, within the hours above specified, to signify the same, that they may be attended to in the ordinary course of visiting (cases of sudden illness excepted), they being attended to at all hours.

"4. Subscribers to provide bottles, pots, and bandages, and to send for their medicines.

"Midwifery cases will be charged to subscribers to the above, 10s. 6d. Vaccination included.

"N.B. Mr. Funge has been established in Cheltenham upwards of five years; for three years has held, and still holds, the appointment of surgeon to a very extensive society, and superintends the preparation of all medicines.

"Cheltenham, August, 1843."

Mr. Taplow, in Chuzzlewit, asks who is Co.? and remarks, that although his name is found in every firm, he never had an opportunity of seeing him. He therefore concludes that himself must be the Co. in his own concern. Mr. Funge acts upon the same idea, in the construction of his Penny Medical and Surgical Society, being not only Mr. Funge himself, but the said Society to boot. Mr. Funge is evidently a great reformer. He belongs to the class of Moses and Sons—they tell you to cut down your tailor's bills—he, your Doctor's. But even Moses has not come to a penny, and there Funge has the start of him. Honour to Funge!

